# A retrospective cohort study to compare post-injury admissions for infectious diseases in burn patients, non-burn trauma patients and uninjured people

**DOI:** 10.1186/s41038-018-0120-5

**Published:** 2018-06-12

**Authors:** Janine M. Duke, Sean M. Randall, James H. Boyd, Mark W. Fear, Suzanne Rea, Fiona M. Wood

**Affiliations:** 1 Burn Injury Research Unit, Faculty Health and Medical Sciences, The University of Western Australia, Perth, WA Australia; 2 Centre for Data Linkage, Curtin University, Perth, WA Australia; 3 Burns Service of Western Australia, Fiona Stanley Hospital and Princess Margaret Hospital, Perth, WA Australia

**Keywords:** Burn, Non-burn trauma, No injury, Infectious diseases, Cohort, Population-based

## Abstract

**Background:**

Injury triggers a range of systemic effects including inflammation and immune responses. This study aimed to compare infectious disease admissions after burn and other types of injury using linked hospital admissions data.

**Methods:**

A retrospective longitudinal study using linked health data of all patients admitted with burns in Western Australia (*n* = 30,997), 1980–2012, and age and gender frequency matched cohorts of people with non-burn trauma (*n* = 28,647) and no injury admissions (*n* = 123,399). Analyses included direct standardisation, negative binomial regression and Cox proportional hazards regression.

**Results:**

Annual age-standardised infectious disease admission rates were highest for the burn cohort, followed by the non-burn trauma and uninjured cohorts. Age-standardised admission rates by decade showed different patterns across major categories of infectious diseases, with the lower respiratory and skin and soft tissue infections the most common for those with burns and other open trauma. Compared with the uninjured, those with burns had twice the admission rate for infectious disease after discharge (incident rate ratio (IRR), 95% confidence interval (CI): 2.04, 1.98–2.11) while non-burn trauma experienced 1.74 times higher rates (95%CI: 1.68–1.81). The burn cohort experienced 10% higher rates of first-time admissions after discharge when compared with the non-burn trauma (hazard ratio (HR), 95%CI: 1.10, 1.05–1.15). Compared with the uninjured cohort, incident admissions were highest during the first 30 days after discharge for burns (HR, 95%CI: 5.18, 4.15–6.48) and non-burn trauma (HR, 95%CI: 5.06, 4.03–6.34). While incident rates remained high over the study period, the magnitude decreased with increasing time from discharge: burn vs uninjured: HR, 95%CI: *30 days to 1 year*: 1.69, 1.53–1.87; *1 to 10 years*: 1.40, 1.33–1.47; *10 years to end of study period:* 1.16, 1.08–1.24; non-burn trauma vs uninjured: HR, 95%CI: *30 days to 1 year*: 1.71, 1.55–1.90; *1 to 10 years:* 1.30, 1.24–1.37; 10 *years to end of study period*: 1.09, 1.03–1.17).

**Conclusions:**

Burns and non-burn trauma patients had higher admission rates for infectious diseases compared with age and gender matched uninjured people. The pattern of annual admission rates for major categories of infectious diseases varied across injury groups. Overall, the burn cohort experienced the highest rates for digestive, lower respiratory and skin and soft tissue infections. These results suggest long-term vulnerability to infectious disease after injury, possibly related to long-term immune dysfunction.

## Background

Traumatic injury continues to be an important cause of morbidity and mortality in both developing and developed nations [1, 2]. Injuries trigger a range of systemic responses, including inflammatory, immune and neuroendocrine responses, which can persist for a long period after the initial injury [3–5]. As part of these host responses, the immune system homeostasis is disturbed and predisposes patients to infections and inflammatory complications during the acute phase of the injury [6, 7]. The induced changes in the immune system can be classified as pro-inflammatory, primarily driven by the innate immune system, and counter-inflammatory, regulated by the adaptive immune system [8]. Clinically, these responses are referred to as systemic inflammatory response syndrome (SIRS), compensatory anti-inflammatory response syndrome (CARS) and mixed effects anti-inflammatory response syndrome [9].

While a growing body of work indicates that serious injury suppresses immune function [8, 10–12] recent animal-based research found long-term immune dysfunction after non-severe burn injury [13]. These results were supported by our previous study, which found increased infectious disease morbidity among burn patients after both minor and severe burns, for a prolonged period after discharge, when compared with an age gender matched cohort of uninjured people [14]. In this study, burn patients were found to have significantly elevated rates of first-time infectious disease admissions after discharge with rates reducing in magnitude from five times higher during the first 30 days post-discharge, 1.7 times higher for the remainder of the first year and 1.4 times higher for the following 9 years. Respiratory, skin and soft tissue and gastrointestinal infections were the most commonly observed primary reason for a post-injury infectious disease hospital admission in this burn cohort [14].

Evidence that sepsis and different types of traumatic injury can initiate different systemic responses [15–17] is growing; however, limited information exists on persistence of these responses after different types of injury and health effects among injury survivors. The objective of this study was to build on previous research and examine potential differences in temporal patterns and risk of post-injury infectious disease hospital admissions associated with different types of injury. We undertook a population-based retrospective cohort study using linked health administrative data to compare post-injury infectious disease admissions, firstly, of patients with burns and non-burn trauma compared with uninjured people, respectively, and secondly, by comparing burn and non-burn trauma patients.

## Methods

This study is part of the Western Australian Population-based Burn Injury Project (WAPBIP) and is approved by the ethics committees of the Western Australian Department of Health (DOHWA) and the University of Western Australia. Linked de-identified hospital and death data were extracted and supplied to researchers by the DOHWA Data Linkage Branch [18]. Methods have been published previously [19].

This study used linked hospital admissions (Hospital Morbidity Data System) and death data of all patients hospitalised for a first burn in Western Australia during 1980–2012 and two comparison cohorts (non-burn trauma patients ~ 1:1; non-injured people ~ 4:1) that were age and gender frequency matched to the burn case for each year of the study and from the same geographic statistical local area. The non-burn trauma cohort was randomly selected by DOHWA Data Linkage Branch and excluded those admitted for burns, effects of foreign bodies entering through orifices, injuries to nerves and spinal cord, poisoning, toxic effects of non-medical substances (e.g. alcohol) and complications of surgical and medical care. The non-injured cohort was randomly selected from the general population (birth registration or electoral roll) and included those who did not have an injury admission during the study period.

International Classification of Diseases (ICD) codes were used to classify type of injury (burn, non-burn trauma). Total burns surface area percent (TBSA %) was classified as minor (TBSA< 20%), severe (TBSA ≥ 20%) or unspecified TBSA. The International Classification for Injury Severity Score (ICISS) [20] was used to estimate injury severity and was derived using survival risk ratios (SRR) (probability of a patient surviving each single injury). For multiple injuries, ICISS was equal to the product of the SRRs assigned to each injury. The injury severity score was classified as follows: minor ICISS ≥ 0.99, moderate ICISS > 0.941 and < 0.99, and severe ICISS ≤ 0.941 [21, 22]. Baseline comorbidity, based on the Charlson comorbidity index (CCI) [23] with a 5-year look-back (0 CCI = 0; 1 CCI > 0) [24], was generated using hospital data. Quintiles of social disadvantage (Socio-Economic Indices for Areas (SEIFA) [25]) and geographic remoteness and access to services (Accessibility Remoteness Index of Australia (ARIA+) [26]), derived from Australian census data, were assigned to each member of the three cohorts.

An infectious disease admission was defined using the ICD code set of major infectious diseases developed by Baker et al. [27] that extended a set of ICD coding created by the US Centers for Disease Control and Prevention [28, 29].

Chi-square tests were used in univariate analyses, and 5% level of significance was applied. The total number of person-years (PY) of follow-up or risk time was estimated from the final discharge date for the first injury admission and the discharge date for the index burn case was used for the corresponding frequency matched non-injured cohort. The number of annual admissions (total) and summed length of hospital stay (LOS) for infectious diseases during the study were used as outcome measures.

Direct standardisation was used to assess age-adjusted trends in rates of hospitalizations for infectious diseases (total) for the burn, non-burn trauma and uninjured cohorts using the age structure of the Australian population at the 2001 national census [30].

Negative binomial regression modelling was used to compare the number of admissions (combined incident and repeat admissions) and LOS for infectious disease: burn vs non-burn trauma vs non-injured. Covariates were included in all models: socio-demographic (gender, indigenous status, 5-year age group, social disadvantage, remoteness of residence), health factors (pre-existing comorbidity, prior infectious disease admission) and year of study entry (to allow adjustment for time-related treatment and referral patterns).

To ensure validity of our findings, additional analyses comparing infectious disease admissions rates were performed using propensity score matching along with generalised estimating equations (GEE). Two propensity score analyses were carried out, comparing infectious disease admission rates between the burn vs uninjured cohorts, and non-burn trauma vs uninjured cohorts. All cases were included within the propensity analysis. Covariate inclusion in the propensity score analyses were chosen based on published methods [31], with potential covariates only included where propensity scores were balanced between blocks, and covariates were balanced across treatment and comparison groups within blocks. Previous infectious disease hospitalisation (prior 5 years), age category and socioeconomic status quintile were included in the models. A logit model was used to calculate propensity scores, with simple nearest neighbour matching conducted with one neighbour. The average treatment effect on the treated (ATET) was used as the outcome measure. GEE using negative binomial regression models were also carried out comparing the number of infectious disease admissions in 5 years before index burn/non-burn trauma injury to the number of infectious disease admissions in 5 years after burn/injury in these two cohorts, respectively.

Standard negative binomial regression analyses were conducted for subcohorts defined by age at study start (< 18, 18–59, ≥ 60 years), gender, injury severity (TBSA; ICISS) and type (closed fractures, open wounds), decade of study entry (1980–1989; 1990–1999; 2000–2012) with adjustment for follow-up time and prior infectious disease admission status (yes/no) using a 5-year look-back period.

Cox proportional hazards regression was used to compare the effects of burns vs. non-burn trauma on first-time (incident) admissions as well as non-burn trauma vs. non-injured. Covariates included in models as listed above. The proportional hazards assumption was tested [32, 33], and adjusted analyses were conducted using cohorts that excluded any person (in each cohort) with a prior admission for infectious disease, and any person (burn, non-burn trauma) with an additional injury admission (other than index) to reduce possibility of additional injury-induced systemic effects [13].

Attributable risk percentage (AR%) [34] was generated from the adjusted hazard ratio and used to estimate the proportions of first-time post-injury admissions where burn and non-burn trauma were component causes. Analyses were performed using Stata version 12 (StataCorp. LP, College Station, USA).

## Results

### Cohort characteristics

There were 30,997 individuals hospitalised with a first burn injury between January 1980 and June 2012. Males accounted for over two thirds of first burn hospitalisations (68%). The median age at time of burn was 23 years old (interquartile range (IQR), 7–39 years).

Burn injuries ranged in thickness and severity. Full thickness burns were found in 9% of the cohort (*n* = 1108), with 44% having partial thickness burns (*n* = 5321), 15% with erythema (first degree) burns (*n* = 1757) and 34% with unspecified burn thickness (*n* = 4143; multiple burn depths could be recorded for a single individual). Those with severe burns (more than 20% TBSA) made up 2.9% of the cohort (*n* = 911), with 47.9% (*n* = 14,854) with non-severe burns (< 20% TBSA). The remaining 49.1% (*n* = 15,232) had an unspecified TBSA. The majority of those with no specified TBSA had a burn admission earlier in the study period. Those with an unspecified TBSA had a median LOS of 3 days (IQR 1–10 days); this was similar to those with < 20% TBSA burns (median LOS (IQR): 4 days (1–10 days), while those with severe burns generally spent longer in hospital (median LOS (IQR): 25 days (12–48 days)). These results suggested those with an unspecified TBSA generally had less severe burns. A small number of individuals died from their burn injury (0.9%, *n* = 283) during the index admission; a further 11.6% (*n* = 3587) died before the end of the study period.

The anatomical location of the burn injury included the head and neck (21%), the trunk (23%), the upper limbs and hands (42%), the lower limbs and feet (34%), the eyes (7%), the respiratory tract and other internal organs (2%), while for 3% the site was unspecified (an individual could have multiple burn locations coded).

The non-burn trauma cohort contained 28,647 individuals with a hospitalisation for a (non-burn) injury. The most common type of ICD classified injury was fractures (35%, *n* = 9944), followed by open wounds (22%, *n* = 6359), contusions and superficial wounds (11%, *n* = 3029), dislocations and sprains (6%, *n* = 1560), internal organ injuries (4%, *n* = 1062) and amputations (2%, *n* = 568), with other or unspecified injuries accounting for the remaining 20% (*n* = 5715). A small number (0.6%, *n* = 165) died from their injury during the index admission; 9.4% (*n* = 2679) died after discharge and before the end of the study period.

The final uninjured cohort consisted of 123,399 individuals with no record of an injury hospitalisation within the study period. By the end of the study period, 6.9% (*n* = 8566) of this uninjured cohort had died.

Socioeconomic and health status characteristics for the three cohorts are found in Table 1. The burn cohort had a higher proportion of indigenous Australians, higher proportions of those with lower socioeconomic status, those that lived in more regional and remote areas and those with pre-existing comorbidity. The non-burn trauma cohort and the uninjured cohort had the same proportion of males (68%) as the burn cohort, and the same median age of 23 years.

**Table 1:** Baseline demographic and pre-existing health status factors for those with a first-burn injury hospitalisation, and age and gender frequency matched non-burn trauma cohort and non-injured cohort, Western Australia, 1980–2012

Characteristics	No injury *N* (%)	Non-burn trauma *N* (%)	Burn injury *N* (%)	*p* value
Total	123,399	28,647	30,997	
Demographic				
Aboriginality				
Yes	2993 (2.4)	2628 (9.2)	4481 (14.5)	< 0.001
Social disadvantage quintiles^a^				
Quintile 1 (most disadvantaged)	14,597 (12.0)	4854 (17.3)	6579 (21.6)	< 0.001
Quintile 2	28,339 (23.4)	9010 (32.1)	9878 (32.4)	
Quintile 3	22,142 (18.2)	5785 (20.6)	6354 (20.8)	
Quintile 4	21,671 (17.9)	4202 (15.0)	3833 (12.6)	
Quintile 5 (least disadvantaged)	34,609 (28.5)	4226 (15.1)	3857 (12.6)	
Remoteness^b^				
Major city	88,278 (72.8)	15,763 (56.3)	15,810 (51.7)	< 0.001
Inner regional	11,725 (9.7)	2967 (10.67)	3360 (11.0)	
Outer regional	11,653 (9.6)	4261 (15.2)	4958 (16.2)	
Remote	5897 (4.9)	2848 (10.2)	3434 (11.2)	
Very remote	3697 (3.0)	2178 (7.8)	3011 (9.8)	
Health status				
Any comorbidity (CCI ≥ 1)^c^	4691 (3.8)	1863 (6.5)	3131 (10.1)	< 0.001
Injury Category				
Low (ICISS ≥ 0.99)	123,399 (100)	19,646 (68.6)	17,917 (57.8)	< 0.001
Medium (0.941 ≤ ICISS < 0.99)	0	7313 (25.5)	10,326 (33.3)	
High (ICISS < 0.941)	0	1688 (5.9)	2754 (8.9)	

^a^Socio-Economic Indices for Areas (SEIFA) Socio-Economic Disadvantage Quintiles: missing values 1.6% burn, 2.0% injury, and 1.7% no injury

^b^Accessibility Remoteness Index of Australia (ARIA+) remoteness classification: missing values 1.4% burn, 2.2% injury, 1.7% no injury

^c^Comorbidity based on derived Charlson Comorbidity Index (CCI) using a 5-year look-back

*ICISS* International Classification for Injury Severity Score

The median length of follow-up after burn injury was 15.6 years (IQR 7.2–24.3 years), 16.6 years (IQR 8.5–24.9 years) for the non-burn trauma cohort and 16.1 years (IQR 8.1–24.6 years) in the non-injured cohort. In total, there were 485,258 years of follow-up in the burn cohort, 472,072 years of follow-up for the injured cohort, and 2,008,855 years of follow-up for the non-injured cohort.

### Admission rates and summed length of stay

A total of 25,463 infectious disease hospital admissions (primary diagnosis) occurring after burn discharge were identified for the burn cohort, with 16,831 infectious disease admissions in the injured cohort and 31,994 in the uninjured cohort. Frequencies of infectious disease admissions for particular conditions are shown in Table 2 for the three cohorts.

**Table 2:** Number of admissions (%) for infectious diseases classified by subconditions in the burn, injured and uninjured cohorts, 1980–2012

	No injury *N* (%)	Non-burn trauma *N* (%)	Burn injury *N* (%)
Enteric infections	4140 (12.9)	1965 (11.7)	2582 (10.1)
Blood stream infections	423 (1.3)	241 (1.4)	476 (1.9)
Sexually transmitted infections	171 (0.5)	53 (0.3)	114 (0.4)
Neurological and eye infections	415 (1.3)	228 (1.4)	469 (1.8)
Ear infections and upper respiratory tract infections	5063 (15.8)	2306 (13.7)	2704 (10.6)
Lower respiratory tract infections	5363 (16.8)	3566 (21.2)	6000 (23.6)
Heart and circulatory system infections	209 (0.7)	108 (0.6)	114 (0.4)
Digestive tract infections (including liver)	7626 (23.8)	2485 (14.8)	2899 (11.4)
Genitourinary infections	2447 (7.6)	1323 (7.9)	2086 (8.2)
Skin and soft tissue infections	2171 (6.8)	2335 (13.9)	4710 (18.5)
Musculoskeletal infections	263 (0.8)	219 1.3)	346 (1.4)
Neoplasms from infection	672 (2.1)	145 (0.9)	229 (0.9)
Postoperative infections	904 (2.8)	806 (4.8)	1305 (5.1)
Other infections	2127 (6.6)	1051 (6.2)	1429 (5.6)
Total	31,994	16,831	25,463

The total number of days spent in hospital with an infectious disease admission was 159,944 days for the burn cohort, 81,619 days for the injured cohort and 130,369 days for the uninjured cohort. The median length of stay for an infectious disease admission was 3 days for the burn cohort (IQR 1–6 days), 2 days for the injured cohort (IQR 1–5 days) and 2 days for the uninjured cohort (IQR 0–4 days; 0 days corresponds to an admission and discharge on the same day).

**Fig. 1:**
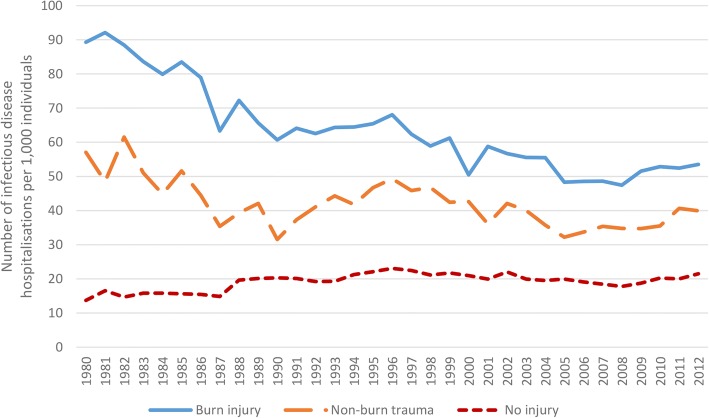
Annual age-standardised rates of admissions for infectious diseases (per 1000 individuals) over time, burn injury vs non-burn trauma vs no injury

Age-standardised rates (ASR) of admissions for infectious diseases over time are found in Fig. 1. Rates were highest for the burn cohort, followed by the non-burn trauma cohort and finally the uninjured cohort. Over the study period, the ASR of infectious disease admissions in the burn cohort decreased by an average annual rate of 1.8% (95%CI: − 2.1 to − 1.6%) while for the non-burn trauma cohort the average annual rate decreased by 1.0% (95%CI: − 1.5 to − 0.6%) and for the uninjured cohort, the rate increased by 0.8% (95%CI: 0.4 to 1.3%).

**Fig. 2:**
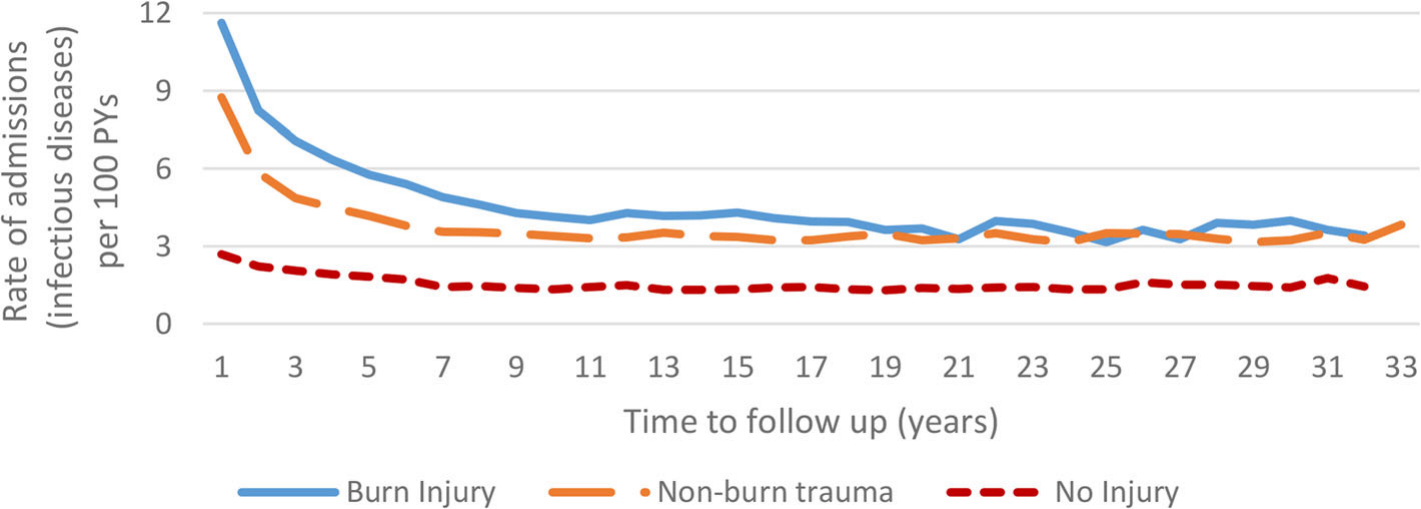
Observed (unadjusted) rates of infectious disease admissions (per 100-person-years) by time since index event burn injury vs non-burn trauma vs no injury

**Fig. 3:**
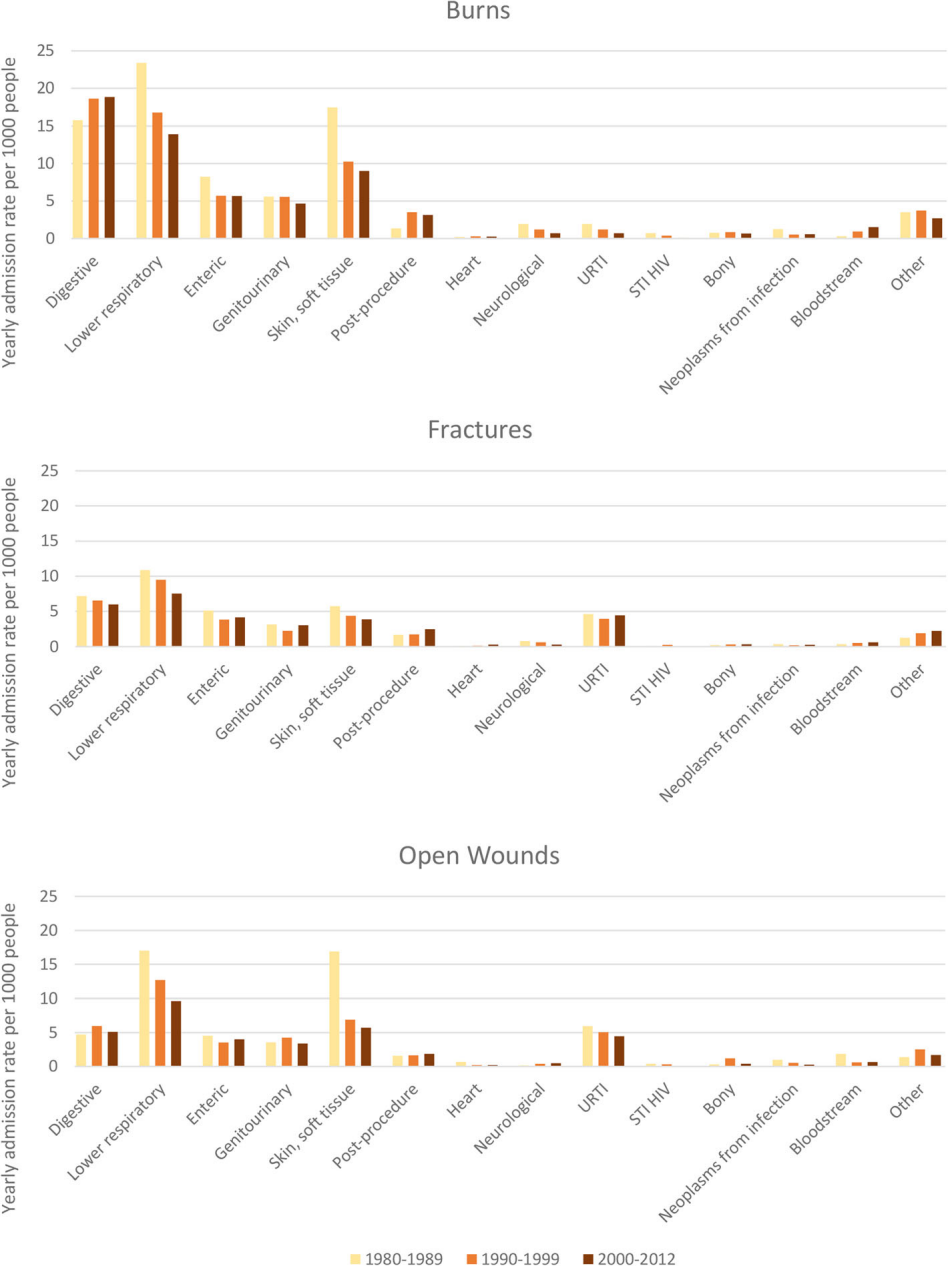
Age-standardised admission rates (per-1000 individuals) for major categories of infectious diseases, by decade of index admission (study entry) for burn injury, fractures and open wounds vs no injury

Observed (unadjusted) rates of infectious disease admissions by time since index event (Fig. 2) identified higher annual rates of infectious disease admissions for the burn and non-burn trauma cohorts compared to the uninjured cohort. This difference was most pronounced in the year after initial injury. Age-standardised hospitalisation rates for major categories of infectious diseases, classified by decade of burn admission (study entry), are presented in Fig. 3.

Adjusted negative binomial analysis, adjusting for socio-demographic and health factors, showed the burn cohort experienced higher rates of infectious disease admissions when compared to the non-burn trauma cohort (IRR, 95%CI: 1.18, 1.14–1.23). This increase was found in both those with a previous infectious disease admission (IRR, 95%CI: 1.27, 1.17–1.38) and those without (IRR, 95%CI: 1.14, 1.10–1.20), and across all time periods (1980–1989: IRR, 95%CI: 1.15, 1.08–1.22; 1990–1999: IRR, 95%CI: 1.16, 1.09–1.25; 2000–2012: IRR, 95%CI: 1.27, 1.16–1.38). The burn cohort spent 1.46 times as long in hospital with infectious diseases (95%CI: 1.30–1.64) when compared with the non-burn trauma cohort. Higher rates of hospital admissions were found in those who sustained their burn when between 18 and 60 years of age compared to the non-burn trauma cohort (IRR, 95%CI: 1.33, 1.25–1.40); no difference was found between these two cohorts for those age under 18 years (IRR, 95%CI: 1.03, 0.97–1.10) and those over 60 years (IRR, 95%CI: 1.11, 0.99–1.25) at the time of injury.

Results of propensity score matching found the average treatment effect on the treated of 0.50 (95%CI: 0.45–0.55) for those with burn as compared to uninjured individuals. Smaller increases were found comparing the injured cohort to the uninjured cohort (ATET, 95%CI: 0.29, 0.25–0.34). Analysis of within-subject differences showed an increase rate of admissions after burn injury as compared with before burn injury (1.16, 1.11–1.21); similar effects were found in the injured cohort (IRR, 95%CI: 1.25, 1.19–1.32).

**Fig. 4:**
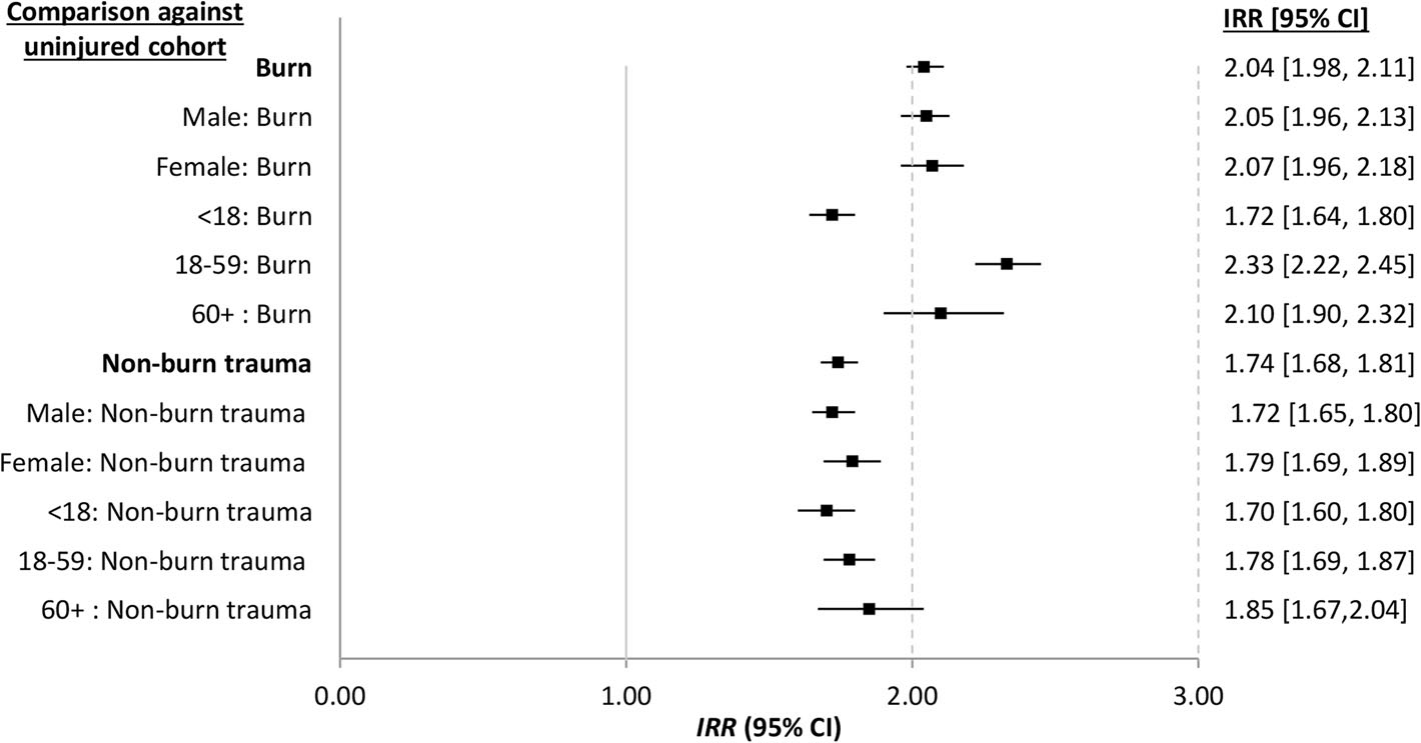
Adjusted incidence rate ratio (IRR) and 95% confidence interval (CI) for infectious disease admissions for age and gender subgroups of burn injury and non-burn trauma compared with no injury

**Fig. 5:**
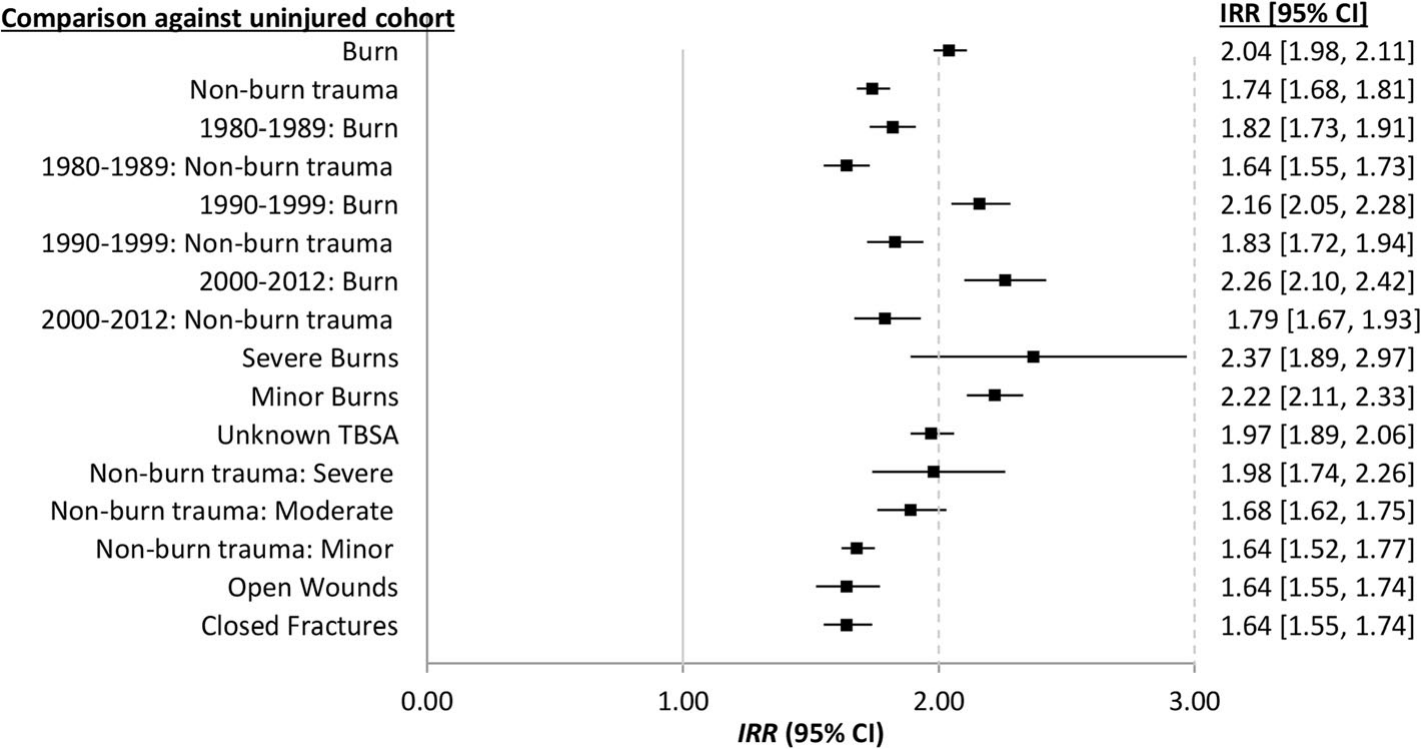
Adjusted incidence rate ratio (IRR) and 95% confidence interval (CI) for infectious disease admissions by decade of index admission, injury type and injury severity, compared with no injury

Analyses comparing the burn and non-burn trauma subcohorts to the respective uninjured subcohorts with respect to age and gender are presented in Fig. 4; study entry and injury severity and type are found in Fig. 5.

Additional analysis was performed investigating the time until first admission for an infectious disease, excluding those in each cohort who had a previous infectious disease hospitalisation, or those with the record of another injury preceding or succeeding the index event. There were 16,758 individuals in the reduced burn cohort, 17,554 in the reduced injured cohort and 112,021 in the reduced uninjured cohort.

The results of adjusted Cox regression modelling showed higher rates of first-time infectious disease admissions for the burn and non-burn trauma cohorts compared with the uninjured cohort, and first-time admission rates were 10% higher in the burn cohort when compared to the non-burn trauma cohort (HR, 95%CI: 1.10, 1.05–1.15). Evidence of non-proportionality required HR analyses to be split into partitioned time windows: adjusted rates were highest during the first 30 days after discharge from burn and non-burn trauma hospitalisation (burn vs uninjured: HR, 95%CI: 5.18, 4.15–6.48; non-burn trauma vs uninjured: HR, 95%CI: 5.06, 4.03–6.34), generally decreasing in magnitude with increasing time from burn/non-burn trauma, but remaining significantly higher over the entire study period (burn vs uninjured: HR, 95%CI: *30 days to 1 year:* 1.69, 1.53–1.87; *1 to 10 years:* 1.40, 1.33–1.47; *10 years to end of study period*: 1.16, 1.08–1.24; non-burn trauma vs uninjured: HR, 95%CI *30 days to 1 year:* 1.71, 1.55–1.90; *1 to 10 years*: 1.30, 1.24–1.37; 10 *years to end of study period:* 1.09, 1.03–1.17).

In total, results suggest that 1058 (28.3%) first post-burn infectious disease hospitalisations were attributable to burn, while 837 (23.3%) infectious disease hospitalisations after a non-burn trauma were attributable to that injury.

## Discussion

Overall, this population-based study revealed that people with both burn and non-burn trauma serious enough to warrant hospitalisation experience increased rates of hospital admissions for infectious diseases after discharge for a prolonged period of time after discharge. When compared with an age and gender frequency matched cohort of people with no record of injury admission during the study period, burn patients had twice the infectious disease admissions while those with non-burn trauma experienced 1.74 times higher admission rates. Within the burns and non-burn trauma cohorts, there was no difference in infectious disease admission rates with respect to gender when compared with respective uninjured subcohorts. However, adult patients with burns experienced higher post-burn infectious disease admission rates than paediatric burn patients. Interestingly, this same trend was observed for patients with non-burn trauma; however, the overlap of the 95%CI suggests little difference by age.

While the rates were significantly higher for the burn and non-burn trauma cohorts as compared with the uninjured, interestingly, the rates for the injury cohorts did decline over time while the admission rates for the uninjured population increased over time. This result may be related to improved surgical and medical techniques over time, patients receiving management by primary care reducing the need for admission, and/or changes in immune responses over time that are yet to be identified. Trends in increasing infectious disease admissions and burden in the general community over the past decade have been reported across a number of countries [27–29, 35].

Assessment by decade of study entry (index injury admission) indicated statistically significant elevated infectious disease admission rates for each decade with a shift to increased rates for those with burns and non-burn trauma after 1990; however, this increase was more pronounced for those with burns. IRR point estimates suggested increases in severity of injury (burns; non-burn trauma) was associated with increased infectious disease admissions, although there was overlap of the 95%CI. Interestingly, subcohort analyses of closed fractures and other open skin trauma (includes punctures, animal bites, cuts, avulsion, lacerations and traumatic amputations) identified similar adjusted hospitalisation rates for infectious diseases (IRR 1.64). However, these rates were 40% less than that indicated for the burn cohort (IRR 2.04), suggesting different effects on prolonged immune dysfunction dependent on the injury aetiology.

In our previous work [36], we identified digestive tract, lower respiratory and skin and soft tissue infections to be the most common among burn patients, with admission rates decreasing by increasing decade of burn admission for lower respiratory and skin and soft tissue infections. For those with open trauma, annual admission rates were highest for lower respiratory and skin and soft tissue infectious disease admissions. In comparison with the burn cohort, skin and soft tissue infectious admission rates were of similar magnitude for the open trauma cohort. However, the annual admission rates for lower respiratory tract infections were approximately 33% lower (for each decade of injury admission) than the burn cohort; digestive tract infectious disease admissions were three times higher in the burn cohort than the open trauma cohort. For those with closed fractures, lower respiratory infections were the most common infectious disease admissions; however, these annual rates were generally half to that observed for the burn cohort.

These results suggested that trauma may lead to susceptibility to infectious disease, with burns having the greatest impact. This susceptibility may be due to long-term immune suppression. Our previous animal studies showed the impact of burn was more marked than excisional injury of the same extent, with long-term T cell profiles suggesting the development of anergy, exhaustion or deletion tolerance to self-antigens [13]. More recently, we have shown increased susceptibility to respiratory viral infection after burn in an animal model [37]. This was associated with a diminished CD8 response and elevated Natural Killer (NK)/NKT responses, which may be compensatory to the dysfunctional CD8 T cell activation observed. Other insults to the skin have also been shown to induce immune dysfunction, with excessive ultraviolet radiation (UVR) known to induce immune dysfunction in both mice and humans [38]. Ageing also leads to decreased immune function, and both UVR exposure and ageing involve decreased dendritic cell function or number concurrent with decreased T cell function through loss of activation ligands and elevated anti-inflammatory cytokine levels [39, 40]. Therefore, it is possible that burn injury induces similar immune dysfunction, leading to the increased hospitalisation for infectious disease that is observed in this population-based study. However, other alternative causes may exist for the observed increase in infectious disease admission. It is possible that the injury causes direct damage to the tissue and that this damage causes the susceptibility to infection. Therefore, rather than a sustained diminished immune response to infection, the susceptibility is caused by dysfunctional barrier properties, which have previously been observed to be a result of burn injury, even in uninjured skin [41]. Alternatively, changes to the microbiome after injury may also lead to susceptibility to colonisation. Changes to both the skin and intestinal microbiome have both previously been reported after burn injury, and these can increase susceptibility to infection [42, 43]. Further work to understand what causes this susceptibility to infectious disease after burn injury will be important to facilitate the use of alternative interventions, either during the acute phase or after recovery to improve recovery and long-term health after burn injury.

In summary, examination of incident admissions for infectious diseases found both the burn and non-burn trauma cohorts were at significantly increased risk over the study period when compared with the uninjured cohort, with the patients experiencing five times the incidence during the first 30 days after discharge. Time-related patterns were similar for the burn and non-burn trauma cohorts in comparison with the uninjured cohort; however, comparison of the burn cohort with the non-burn trauma cohort identified a 10% higher rate of incident infectious disease admissions for those with burns. From the incident analyses (Cox regression), we were also able to estimate that 28% (*n* = 1058) and 23% (*n* = 837), respectively, of first-time infectious disease admissions could be attributed to the initial burn and non-burn trauma event and prevented in the absence of the injury.

### Strengths and limitations

Limited long-term data of injury patients, burns and other non-burn traumatic injury, are available and/or published, particularly in regard to infectious diseases. The strengths of this study are related to the use of population-based linked health data with a median follow-up time of approximately 15 years (minimum > 0; maximum 33 years) and the use of a published and comprehensive list of ICD-classified infectious diseases [27–29]. Out-of-state migration in Western Australia is relatively low at less than 2.8% per annum, and as such loss-to-follow-up is not likely to cause systematic error in this population-based study [44]. We were able to adjust for cohort differences in socioeconomic disadvantage, Aboriginality, comorbidity and geographic distance/access to services, including medical services and hospitals [45–47] that may be associated with infectious disease admissions, by including these variables in the multivariable regression analyses [34]. The census-derived socioeconomic disadvantage variable used in this study has high correlation with nutrition status, alcohol consumption, smoking and physical activity, factors that may also affect infectious disease status [48–50]. ICD codes were used to classify disease, injury and injury severity and applied to each cohort, and as such, any misclassification would be expected to lead to underestimates of the infectious disease outcomes measured. Hospital administrative data are characteristically not collected for research purposes. Our analyses are limited by the variables available in the datasets. Other clinical data (including smoking, diet, alcohol use, primary care attendance, pathology and medications) were not included in these health administrative datasets, and it is possible that a level of residual confounding may exist.

To ensure validity of our conclusions, our key hypothesis was tested using a range of statistical methods, each with their own strengths and weaknesses. The use of standard multivariate regression analysis comparing cohorts allowed us to include the full gamut of potential confounding factors available to us. Propensity score analysis allowed us to account for imbalances in covariates between cohorts, although only a minimal number of covariates can be concluded. Finally, GEE methods measuring within-subject differences before and after the injury event avoid issues of comparability between cohorts, but as a limitation may be subject to age-based cohort effects. The fact that all methods pointed towards the same findings provides additional strength in our conclusions.

## Conclusions

These results showed that injury patients with burns and non-burn trauma had increased admission rates for infectious diseases for a prolonged period after discharge for their initial injury. Burn patients had significantly higher infectious disease admission rates when compared with patients with other types of trauma and uninjured people. Injury to the skin, burn and other open wounds were associated with significantly higher rates of skin and soft tissue infections for an extensive period after discharge. Lower respiratory tract infectious disease admissions were also higher for those with burns and open wounds when compared to those with closed fractures, while burn patients had higher rates of digestive infections. The post-injury infectious disease patterns suggest that different immune and inflammatory responses are triggered by injury to the skin, and immune dysfunction may persist for a prolonged period after injury. These results also suggest that it may be prudent to consider how a patient’s immune system might be supported after discharge.

## Abbreviations

95%CI: 95% confidence interval
AR%: Attributable risk percent
ARIA+: Accessibility Remoteness Index of Australia
ASR: Age-standardised rates
ATET: Average treatment effect on the treated
DOHWA: Department of Health, Western Australia
GEE: Generalised estimating equations
HR: Hazard ratio
ICD: International Classification for Diseases
ICISS: International Classification for Injury Severity Score
IQR: Interquartile range
IRR: Incidence rate ratio
LOS: Length of stay
PY: Person-years
SEIFA: Socio-Economic Index for Areas
SRR: Survival risk ratio
TBSA: Total body surface area
UVR: Ultraviolet radiation
WAPBIP: Western Australian Population-based Burn Injury Project
